# An Amperometric Biosensor for Uric Acid Determination Prepared From Uricase Immobilized in Polyaniline-Polypyrrole Film

**DOI:** 10.3390/s8095492

**Published:** 2008-09-04

**Authors:** Fatma Arslan

**Affiliations:** Department of Chemistry, Faculty of Arts and Sciences, Gazi University, 06500 Ankara, Turkey; E-mails: fatma@gazi.edu.tr, f-arslan@windowslive.com; Tel.: +90 (312) 2021518; Fax: +90 (312) 2122279

**Keywords:** Uric acid, Uricase, Biosensor, Polypyrrole, Polyaniline

## Abstract

A new amperometric uric acid biosensor was developed by immobilizing uricase by a glutaraldehyde crosslinking procedure on polyaniline-polypyrrole (pani-ppy) composite film on the surface of a platinum electrode. Determination of uric acid was performed by the oxidation of enzymatically generated H_2_O_2_ at 0.4 V vs. Ag/AgCl. The linear working range of the biosensor was 2.5×10^-6^ × 8.5×10^-5^ M and the response time was about 70 s. The effects of pH, temperature were investigated and optimum parameters were found to be 9.0, 55 °C, respectively. The stability and reproducibility of the enzyme electrode have been also studied.

## 1. Introduction

Uric acid is an end product from purine derivatives in human metabolism. The assay of uric acid in body fluids (e.g. serum and urine) is a clinically valuable diagnostic indicator [1]. The presence of elevated uric acid levels is a sign of gout, hyperuricemia, or Lesch-Nyhan syndrome [2]. Similarly, elevated uric acid levels are related to other conditions including increased alcohol consumption, obesity, diabetes, high cholesterol, kidney disease, and heart diseases. Many epidemiological studies have suggested that serum uric acid is also a risk factor for cardiovascular disease [3].

Uricase, which is detnoted as Uox, catalyzes *in vivo* oxidation of uric acid in the presence of oxygen as an oxidizing agent, producing allantoin and CO_2_ as the oxidation products of uric acid and hydrogen peroxide as the reduction product of the O_2_:

$$\text{uric acid}+{\text{O}}_{2}+{\text{H}}_{2}\text{O}\overset{\text{uricase}}{\to }\text{allantoin}+{\text{CO}}_{2}+{\text{H}}_{2}{\text{O}}_{2}$$

The amperometric determination of uric acid can be made by electrochemically oxidizing the produced H_2_O_2_ [4]:

$${\text{H}}_{2}{\text{O}}_{2}\to {\text{O}}_{2}+2{\text{H}}^{+}+2{\text{e}}^{-}$$

There are some studies on enzymatic sensors based on detection of hydrogen peroxide [5-7].

Conducting polymers (polypyrrole or polyaniline) have been used as a matrix for immobilizing enzymes [4, 8-12]. A literature search revealed that there were no reports of biosensors prepared by the immobilization of uricase enzyme on a polyaniline-polypyrrole film. Therefore, a new amperometric biosensor for the determination of uric acid has been developed by immobilizing uricase on a polyaniline-polypyrrole film. The optimum working conditions (pH, temperature) of biosensor were investigated. In addition, the factors (storage stability, reproducibility) effecting biosensors working performance were investigated.

## 2. Result and Discussion

In this study a new uric acid sensitive amperometric enzyme electrode was prepared by the use of conductive polymer polyaniline-polypyrrole. The parameters effecting to the performance of the biosensor were investigated.

### 2.1. The working potential

**Figure 1.**
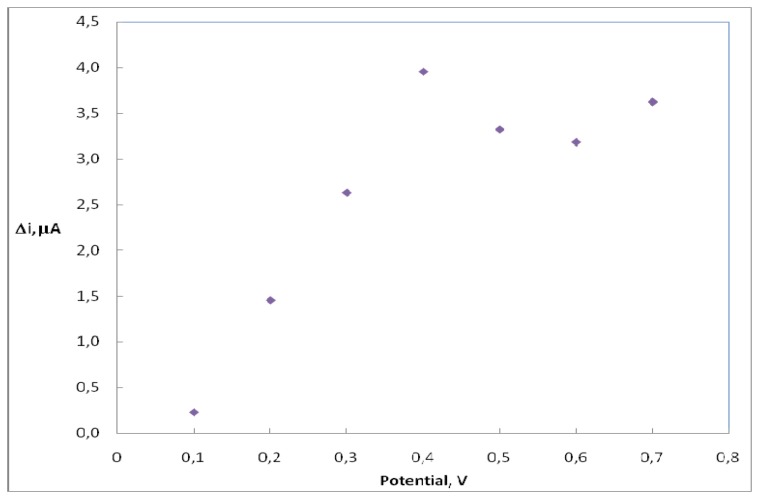
The oxidation of hydrogen peroxide (0.1 mM) at different potential using Pt/Pani-ppy electrode.

Current differences of H_2_O_2_ (0.1 mM) in different potentials (0.1, 0.2, 0.3, 0.4, 0.5, 0.6, 0.7 V) were measured by using pt/pani-ppy electrode and plotted against potential. In Figure 1, it is shown that the current of H_2_O_2_ increases linearly until a potential of 0.4V. After 0.4 V a deviation from linearity was observed. Therefore 0.4 V was used as working potential.

### 2.2. The effect of pH

**Figure 2.**
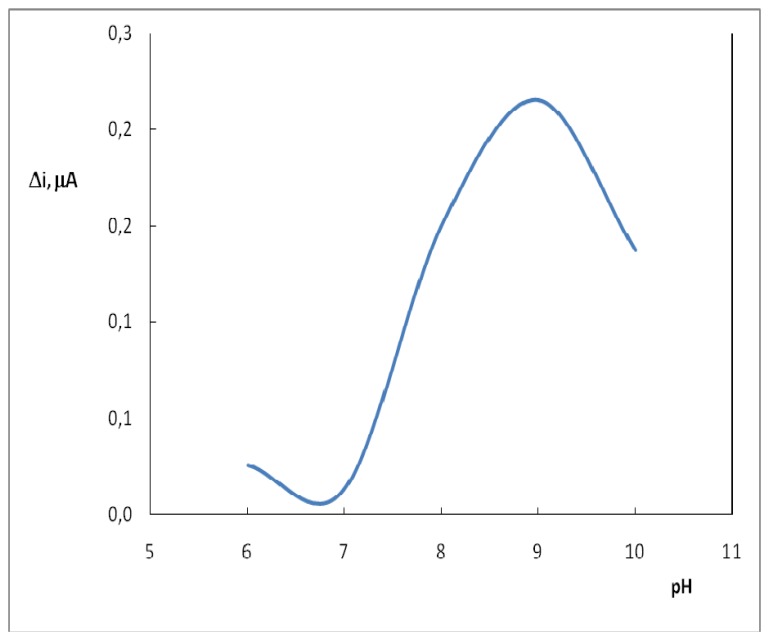
The effect of solution pH on the response to 4.0×10^-5^ M uric acid in 0.05 M borate buffer solution of 25 °C. The operating potential is 0.4 V vs. Ag/AgCl.

Buffers at various pH values were tested to investigate the effect of pH. The pH of the buffers was varied between 6.0 and 10.0. The measurements were performed at a constant uric acid concentration of 4.0×10^-5^ M. Figure 2 shows that the maximum response was obtained at pH 9.0. In uric acid determination with determination with biosensors, pH values other than 9.0 were employed in literature (pH 7.5, 8.0) [4]. This was attributed to the fact that the used polymer and the type of immobilization were different [13].

### 2.3. The effect of Temperature

**Figure 3.**
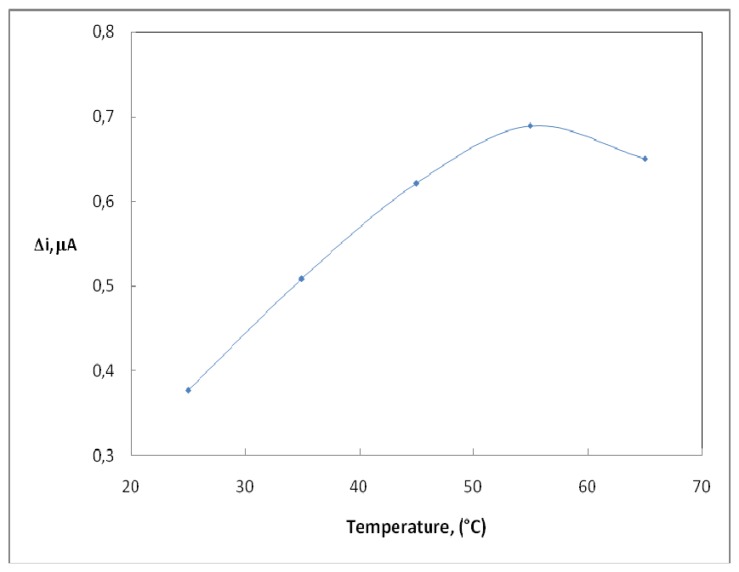
The effect of temperature upon the sensitivity of biosensor against 4.0×0.10^-5^ M uric acid (0.05 M, pH 9.0 borate buffer).

Temperature has a great effect on enzyme activity and it is important to investigate the temperature dependence of the response of the enzyme electrode. The temperature influence on the response of uric acid enzyme electrode was tested between 25°C and 65°C at pH 9.0 using constant uric acid concentration of 4.0×10^-5^ M (Figure 3). As seen from the figure, the electrode response increases with temperature up to 55 °C and decreases afterwards. The highest electrode response was obtained at 55 °C. The sudden decrease in the responses after 55 °C is thought to be caused by the denaturation of the enzymes. The study was carried out at 25°C due to the difficulties involved in working at 55 °C. Similar situations with other enzyme electrodes were encountered in the literature [14].

### 2.4. Substrate Concentration and Calibration Curves

**Figure 4.**
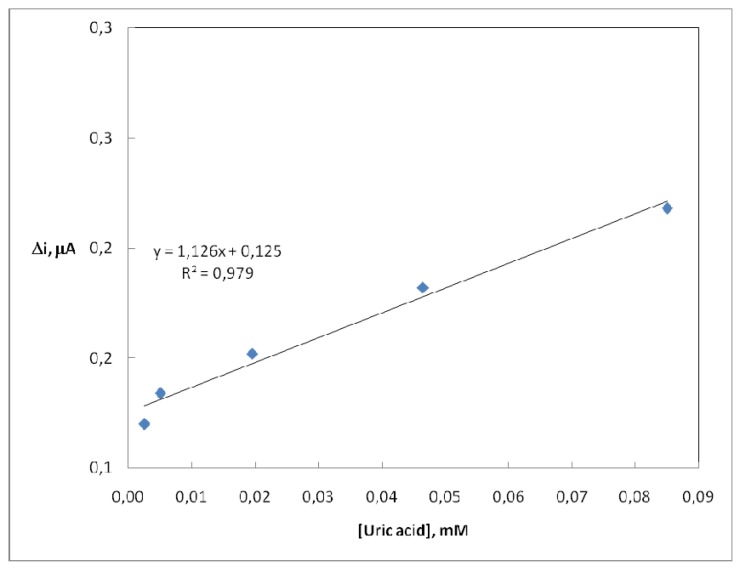
The calibration curve of uric acid biosensor (0.05 M, pH 9.0 borate buffer, 25°C).

There are linear parts ranging between 2.5×0.10^-6^- 8.5×0.10^-5^ M (R^2^: 0.979). The graph for the calibration curve is given in Figure 4.

**Figure 5.**
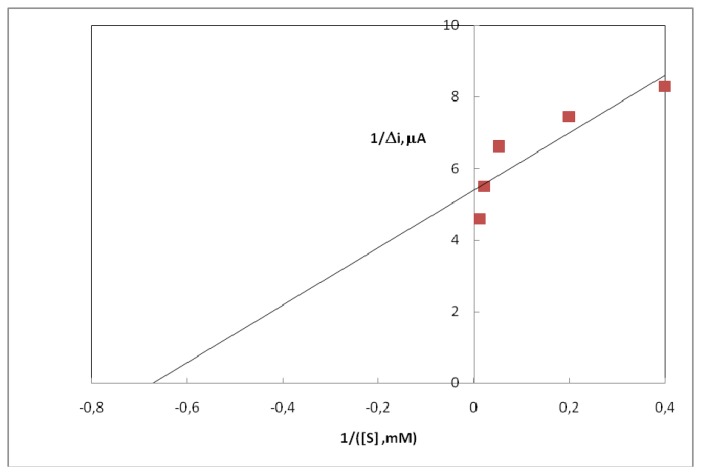
The effect of uric acid concentration upon the amperometric response of free and immobilized uricase (Lineweaver-Burk Plot).

It has been shown that the linearity of graph is highly satisfactory and it can be used for the quantitative determination of uric acid. The low detection limit of the biosensor prepared was found to be 1.0×10^-6^ M. The K_m(app)_ constant, a specific parameter of enzymes, was computed by the use of 1/[uric acid]-1/v graph (Lineweaver-Burk plot) (Figure 5). K_m(app)_ value was found as 1.57 mM.

The K_m(app)_ value, which shows the affinity of the biosensors, was 1.57 mM and the values cited in literature were 0.170 mM and 0.90 mM [15,16]. Since the enzymatic reactions take place through a carrier medium, the affinity of the enzyme towards the substrate is not the same in every carrier media. Therefore it is considered normal that K_m (app)_ values should differ.

### 2.5. The Reproducibility of the Enzyme Electrode

**Figure 6.**
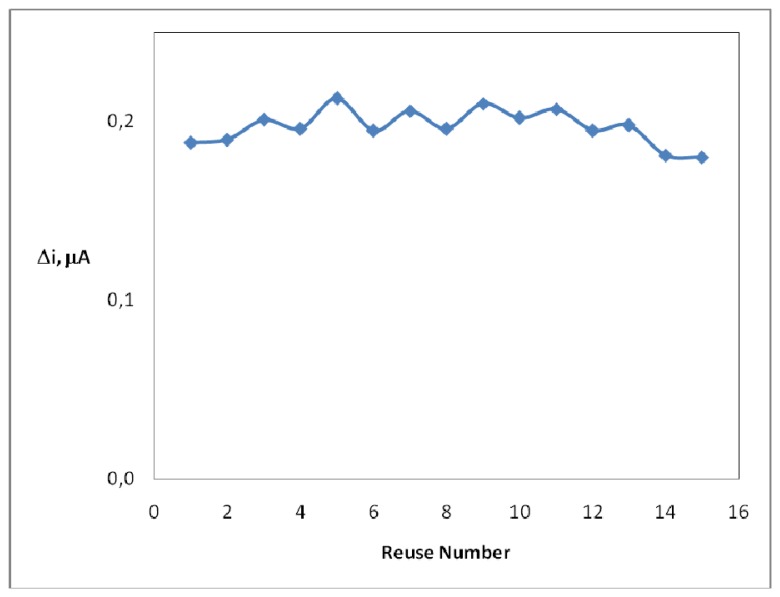
Reuse number of the enzyme electrode (0.05 M, pH 9.0 borate buffer, 25 °C).

In order to test the reproducibility of the enzyme electrode prepared, the current changes obtained after subsequent usage were plotted against the number of measurements. It was observed that 4% of the initial amperometric response decreased after 15 measurements at a constant uric acid concentration of 2.7×10^-5^ M (Figure 6). This result indicates that 4% of initial activity of the immobilized enzyme reduced. This shows that the reproducibility of the uric acid electrode was highly satisfactory.

### 2.6. Storage Stability

**Figure 7.**
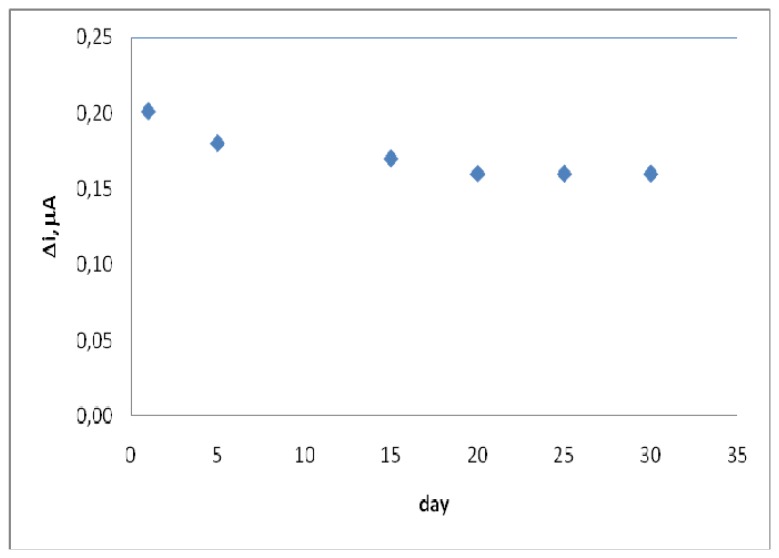
Storage stabilization of the enzyme electrode (0.05 M, pH 9.0 borate buffer, 25°C).

The response of the enzyme electrode prepared under optimum condition was measured for a period 4 weeks at constant uric acid concentration (2.7×10^-5^ M). The result of 5 measurements during this period is plotted in Figure 7. It was observed that 20% of the initial amperometric response decreased at the end of the 4^th^ week. This result shows that 20% of initial activity of the immobilized enzyme reduced. This indicates that the biosensor prepared can be used for quite a long time.

### 2.7. Interference Effect

A few common substances found in serum or urine was studied for any possible interfering effects on the analysis of uric acid. Known concentrations of ascorbic acid, glucose, bilirubin, urea, paracetamol (acetaminophen), were added to 4.0×10^-5^ M uric acid solution and the results are shown in Table 1. It has been shown that glucose, urea, bilirubin and ascorbic acid have been no interfering effects on the analysis of uric acid. But interference of paracetamol (31%) in the analysis of uric acid has been observed.

## 3. Experimental Section

### 3.1. Equipment and Reagents

The electrochemical studies were carried out using an Epsilon EC electrochemical analyzer with a three-electrode cell. The working electrode was a Pt plate (0.5 cm^-2^). The auxiliary and the reference electrodes were a Pt wire and Ag/AgCl, respectively. The pH values of the buffer solutions are measured with ORION Model 720 a pH/ionmeter. Temperature control was achieved with Grant W14 thermostat. Uricase (EC 1.7.3.3, purified from the microorganism and with an activity of 10 unit.mL^-1^) and uric acid were purchased from Sigma. Pyrrole and aniline were supplied from Fluka. All other chemicals were obtained from Sigma. All the solutions were prepared using distilled water.

### 3.2. Preparation of Uric Acid Biosensor

The surface of the Pt plate electrode was mechanically polished using 1 μm alumina powder on a polishing pad. The polished electrode was rinsed to remove alumina with a sharp stream of water, and then ultrasonically treated in a mixed solution of 2 M HCl-alcohol (50%, V/V) for 15 min. Finally the electrode was washed with water and dried at room temperature [17].

The electrode was immersed in a solution of 0.1 M aniline in 0.1 M H_2_SO_4_ (10 mL). The solution was purged with nitrogen in order to remove the oxygen. The electropolymerization of aniline upon the electrode surface was achieved by the cyclic voltammetric scans between 0.0 and 0.9 V at a scan rate of 50 mV/s (5 cycles). The electrode was washed with distilled water after the coating process. The pani electrode was immersed in a solution of 0.1 M pyrrole in 0.1 M H_2_SO_4_ (10 mL). The solution was purged with nitrogen in order to remove the oxygen. The electropolymerization of pyrrole upon the polyaniline electrode surface was achieved by the cyclic voltammetric scans between 0.0 and 0.9 v at a scan rate of 50 mV/s (5 cycles). The electrode was washed with distilled water after the coating process [18].

Uricase enzyme (10 unit/mL, 50 μL) was dropped on surface of platinium/polyaniline-polypyrrole (Pt/Pani-Ppy) electrode and dried room temperature. Glutaraldehyde solution (% 0.05 m/v, 15 μL) was dropped on the pani-ppy-enzyme layer, washed with borate buffer (0.05 M, pH: 9) and dried at room temperature. The enzyme electrode was kept in refrigerator at +4 °C in borate buffer when it was not in use.

### 3.3. Amperometric Measurements

In order to determine whether the enzyme electrode was sensitive to uric acid, an aqueous buffer solution containing 0.05 M borate at a pH of 9.0 was added to the cell. The solution, a steady-state background current (i_a)_, was allowed to decay at a constant value at 0.4 V. Then the aliquots uric acid stock solution was added to the cell using a micropipette, and stirred for 10 min. The response current (i_b_) of the biosensor against uric acid was measured after 200 s following the application of a constant potential of 0.4 V (vs. Ag/AgCl) by electrochemical oxidizing the produced H_2_O_2_. The current values (Δi= i_b_- i_a_) were plotted.

## 4. Conclusions

In conclusion, the uric acid biosensor prepared in this study:

- Is usable in a large concentration range (2.5×0.10^-6^-8.5×0.10^-5^ M).
- Has a very low detection limit 1.0×0.10^-6^ M.
- Has an acceptable response time for a biosensor (70 second).
- Gives highly reproducible results (the electrode lost 4% of the initial amperometric response at the end of the 15^th^ measurement).
- Has a satisfactory storage stabilization (the electrode lost 20% of the initial amperometric response at the end of the 4^th^ week).
- The Km (app) value of uricase enzyme immobilized in polyaniline-polypyrrole film was 1.57 mM.

## Figures and Tables

**Table 1.** Interfering substances (constant uric acid concentration 4.0×10^-5^ M) on the amperometric response of the biosensor.

**Interfering substances**	**Concentration**	**Interference effect of Substances**
Ascorbic acid	1.0×10- M	-
Glucose	5.0×10- M	-
Bilirubin	1.0×10- M	-
Urea	1.0×10- M	-
Paracetamol	1.0×10- M	31%
